# Particle Size and Biological Fate of ZnO Do Not Cause Acute Toxicity, but Affect Toxicokinetics and Gene Expression Profiles in the Rat Livers after Oral Administration

**DOI:** 10.3390/ijms22041698

**Published:** 2021-02-08

**Authors:** Jin Yu, Soo-Jin Choi

**Affiliations:** Division of Applied Food System, Major of Food Science and Technology, Seoul Women’s University, Seoul 01797, Korea; ky5031@swu.ac.kr

**Keywords:** zinc oxide nanoparticles, particle size, biological fate, toxicokinetics, transcriptomics

## Abstract

Zinc oxide (ZnO) particles have been used as dietary supplements because zinc is an essential trace element for humans. Along with the rapid development of nanotechnology, the use of ZnO nanoparticles (NPs) is increasing in the food industry, but their oral toxicity potential still remains to be answered. In this study, the effects of particle size and biological fate of ZnO on acute toxicity, toxicokinetics, and gene expression profiles in the livers were investigated after oral administration of ZnO NPs (N-ZnO), bulk-sized ZnO (B-ZnO) or Zn ions in rats. The plasma concentration-time profiles after a single-dose oral administration of ZnOs differed depending on particle/ionic forms and particle size, showing high absorption of Zn ions, followed by N-ZnO and B-ZnO, although in vivo solubility did not differ from particle size. No significant acute toxicity was found after oral administration of ZnOs for 14 days in rats. However, transcriptomic responses in the livers were differently affected, showing that metabolic process and metal biding were up-regulated by Zn ions and N-ZnO, respectively, which were not pronounced in the liver treated with B-ZnO. These findings will be useful to predict the potential oral toxicity of ZnO NPs and further mechanistic and long-term exposure studies are required to assume their safety.

## 1. Introduction

Nanotechnology development in the food industry allows improving color, flavor, and nutritional values, increasing shelf-life, and monitoring integrity of the foods, and may lead to produce food additive nanoparticles (NPs) [1,2,3]. Among food additive particles, zinc oxide (ZnO) is directly added to foods as a zinc (Zn) supplement because Zn is an essential trace element, which plays an important role in enzyme activity, protein and nucleic acid synthesis, hematopoiesis, and neurogenesis [4,5]. The effects of dietary supplementation of ZnO NPs on growth performance compared with conventional ZnO particles have been demonstrated in broilers and weaned pigs [6,7]. Moreover, the administration of ZnO NPs showed significant increases in body growth rate, appetite, and hair growth compared with general bulk-sized ZnO particles in an Zn-deficient rat model [5].

Information about the safety aspect of food additive ZnO NPs is still insufficient, which leads to increase concerns about human health. Although ZnO has been classified as generally recognized as safe [8], ZnO NPs may exhibit unexpected biological responses compared with conventional bulk-sized ZnO due to their different physicochemical properties [9]. Several studies have reported high toxicity of ZnO NPs compared with larger-sized ZnO [10,11,12,13]. Hsiao et al. demonstrated size-dependent cytotoxicity and inflammation responses of ZnO NPs in the human lung carcinoma epithelial cells [10]. ZnO NPs showed higher toxicity than micro-sized ZnO in the human colon cancer cells [11]. More pronounced toxicity of ZnO NPs toward osteoblast cancer cells compared with micro-scaled ZnO and significant alteration in phagocytic capacity of monocytes by ZnO NPs were also demonstrated [12,13].

Another important factor to be considered is the interactions between NPs and biomolecules in physiological environments [14,15,16,17], which may change the physicochemical property of NPs. In particular, the formation of particle-protein corona easily occurs after injection or oral ingestion, which can trigger an immune response and affect the fate and toxicity of NPs [14,16]. Our previous proteomic study revealed that ZnO NPs interacted more strongly with rat plasma proteins than bulk-sized ZnO [17,18]. Biological responses, such as cytotoxicity, cellular uptake, intestinal transport, and oral absorption, were also highly affected by the interactions between ZnO NPs and saccharides or food proteins [19,20], suggesting that ZnO NPs may induce unexpected toxicity. On the other hand, NPs encounter a complex matrix condition of the gastrointestinal (GI) tract after oral intake [16]. The environment of GI fluids such as pH, ionic strength, and composition can alter the biological fate of NPs [16]. Several reports demonstrated that ZnO NPs can be easily dissolved under acidic conditions like gastric fluid [17,21,22]. Indeed, the toxic effect of ZnO NPs was reported to be mainly related to Zn ions released from particles [23,24]. These results imply that the biological fate of ZnO NPs can affect their toxicity. Moreover, food additive ZnO NPs can be daily taken up without intention, which cannot exclude their toxicity potential after a long-term exposure. However, no attempt has been made to investigate oral toxicity of ZnO NPs with respect to particle size and biological fate in terms of toxicokinetics and transcriptomics.

Accordingly, the aim of this study was to investigate the effects of particle size (bulk, B-ZnO; nano, N-ZnO) and biological fate (zinc chloride as a Zn ion) of ZnO on in vivo biological systems. Toxicokinetics and in vivo solubility of ZnO particles or Zn ions were evaluated after a single-dose oral administration in rats. Oral toxicity, tissue distribution, and gene expression profiles in the livers were evaluated after repeated oral administration of ZnO particles or Zn ions in rats for 14 days to predict the potential effect of each material on biological functions.

## 2. Results

### 2.1. Characterization of B-ZnO and N-ZnO

The constituent particle sizes, size distributions, and shape of ZnO particles were examined by scanning electron microscopy (SEM) (Supplementary Figure S1). Both particles showed irregular shape, and the constituent particle sizes of B-ZnO and N-ZnO were 268.0 ± 53.0 nm and 86.3 ± 23.8 nm, respectively. The size distribution of N-ZnO was narrower than that of B-ZnO.

The dynamic light scattering (DLS) results show that only particle fraction larger than 200 nm was observed for B-ZnO, whereas N-ZnO had a small portion of particle fraction ranged from 100 to 200 nm (Table 1). The Z-average diameters were much larger than the constituent particle sizes measured by SEM, showing 603.6 ± 1.9 nm and 401.1 ± 4.2 nm for B-ZnO and N-ZnO, respectively. On the other hand, electrophoretic light scattering (ELS) results indicate positive zeta potentials of B-ZnO and N-ZnO, showing 9.5 ± 0.4 and 12.8 ± 0.2 mV, respectively.

### 2.2. In Vivo Dissolution Properties of B-ZnO and N-ZnO

**Figure 1:**
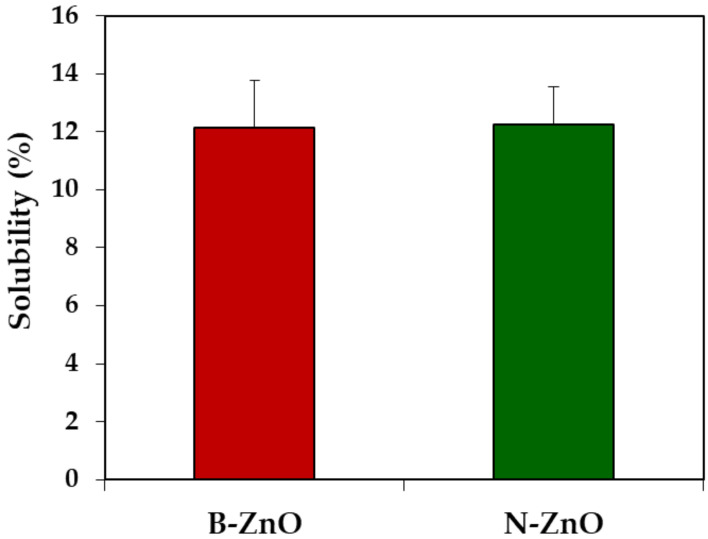
In vivo dissolution properties of ZnO particles in the gastric fluid at 15 min post-oral administration in rats. No significant difference between B-ZnO and N-ZnO was found (*p* > 0.05).

In vivo solubility of ZnO particles was evaluated after a single-dose oral administration of ZnO particles (100 mg/kg) in rats, followed by collecting the gastric fluid. As shown in Figure 1, in vivo solubilities of both B-ZnO and N-ZnO were ~12% without significant difference between particle sizes (*p* > 0.05). In other words, most ZnO particles remained as particle forms in the gastric fluid after oral administration, regardless of particle size.

### 2.3. Toxicokinetics

**Figure 2:**
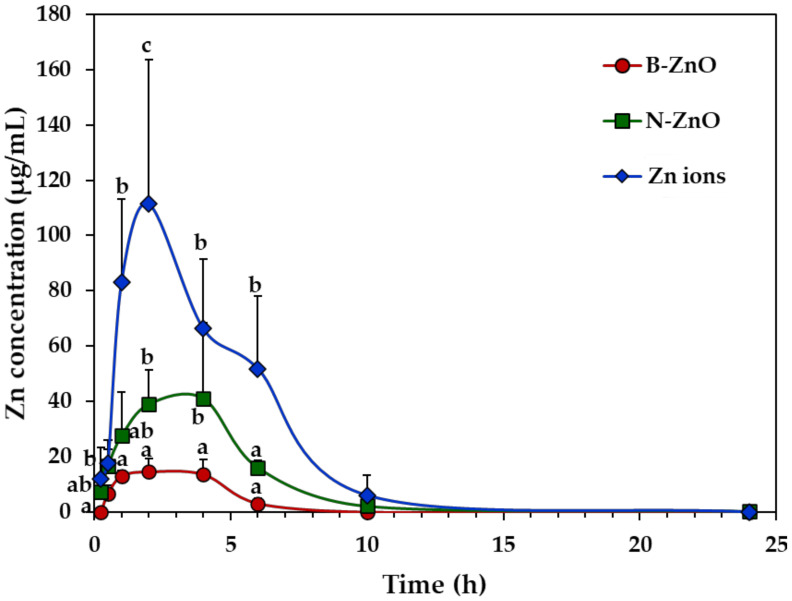
Plasma concentration–time profiles after a single-dose oral administration of B-ZnO, N-ZnO or Zn ions in rats. a, b, and c indicate significant differences among B-ZnO, N-ZnO, and Zn ions (*p* < 0.05).

The plasma concentration–time profiles of ZnO particles (100 mg/kg) or an equivalent amount of Zn ions were evaluated after a single-dose oral administration in rats, by measuring total Zn levels in the plasma using inductively coupled plasma–atomic emission spectroscopy (ICP-AES). Figure 2 shows different toxicokinetic profiles depending on the administered materials. The peak concentration of Zn ions was remarkably higher than those of ZnO particles (B-ZnO and N-ZnO), and significantly higher plasma level of N-ZnO than that of B-ZnO was found. In all cases, increased plasma Zn concentrations decreased to zero levels within 10 h after oral administration.

Toxicokinetic parameters reveal the highest C_max_, AUC values, and oral absorption (%) of Zn ions, followed by the order of N-ZnO and B-ZnO (Table 2). T_max_ values for Zn ions and ZnO particles were 1.6 h and ~3.0 h, respectively. When toxicokinetic profiles between particle sizes were compared, N-ZnO had significantly higher C_max_, AUC values, and oral absorption than those for B-ZnO. In particular, oral absorption of N-ZnO (14.5%) was higher than that of B-ZnO (5.0%), although these values were much less than that of Zn ions (31.4%). Short half-life and residence time of both ZnO particles and Zn ions were also confirmed by low T_1/2_ and MRT values.

### 2.4. Oral Toxicity and Tissue Distribution

**Figure 3:**
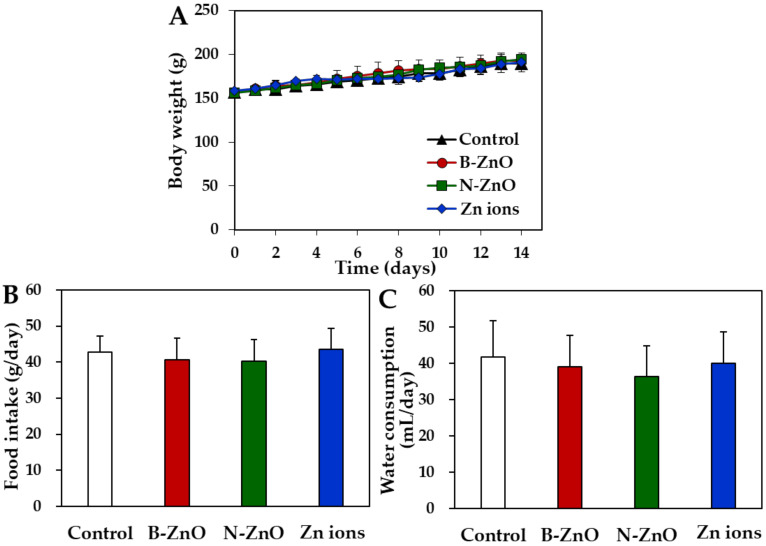
Changes in (**A**) body weight gain, (**B**) food intake, and (**C**) water consumption after 14-day repeated oral administration of B-ZnO, N-ZnO or Zn ions in rats. No significant differences among non-treated control and treated groups (B-ZnO, N-ZnO, and Zn ions) were found (*p* > 0.05).

After repeated oral administration of ZnO particles (100 mg/kg) or an equivalent amount of Zn ions for 14 days in rats, no significant abnormality was found in terms of changes in body weight gain, symptoms, and food intake, and water consumption (Figure 3). The organo-somatic indices, representing the proportion of organs to body weight, show that all organ weights treated with ZnO particles or Zn ions were not significantly affected compared with non-treated control group (Table 3). The dose was chosen based on the previous report, showing that the lowest-observed-adverse-effect level (LOAEL) of ZnO NPs was 125 mg/kg after 90-day repeated oral administration in rats [25]. The dose in the present study was set at 100 mg/kg, which is lower than the LOAEL value, aiming at evaluating transcriptomic analysis at no acute toxic level. Oral administration was carried out for 14 consecutive days because acute toxic effect is observed for 14 days and dose range-finding study for subchronic toxicity test is performed for 14 days. 

**Figure 4:**
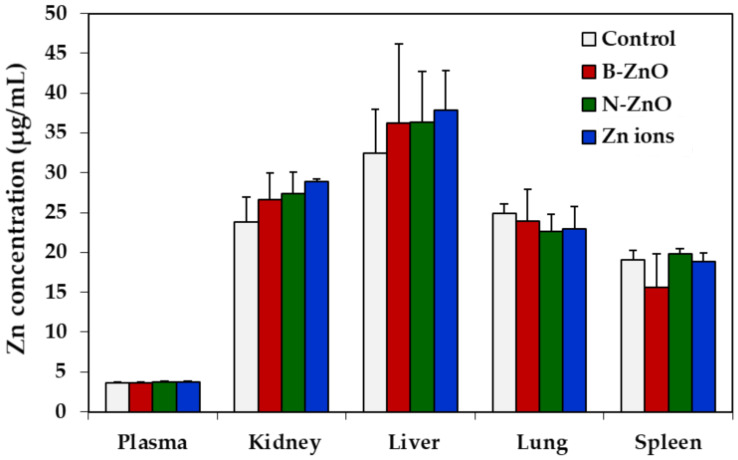
Tissue distribution of B-ZnO, N-ZnO or Zn ions after 14-day repeated oral administration in rats. No significant differences among non-treated control and treated groups (B-ZnO, N-ZnO, and Zn ions) were found (*p* > 0.05).

Tissue distributions of ZnO particles or Zn ions were determined in the plasma, kidney, liver, lung, and spleen by measuring total Zn levels using ICP-AES. The Zn levels of the plasma and rat organs treated with ZnO particles or Zn ions were not significantly elevated compared with non-treated control (Figure 4). In other words, no accumulation of ZnO particles or Zn ions in the main organs was found after 14-day repeated oral administration in rats.

### 2.5. Gene Expression Profiles in the Rat Livers Following 14-Day Repeated Oral Administration

**Figure 5:**
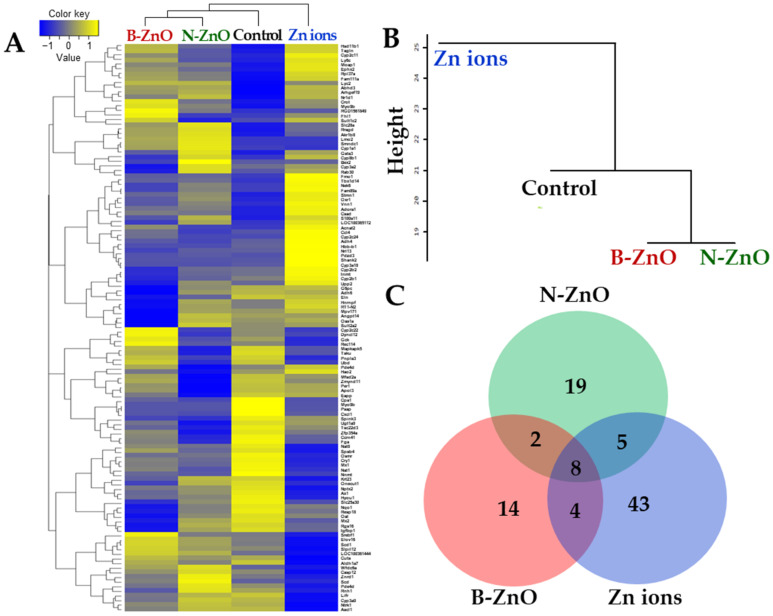
RNA sequencing analysis in the rat livers after 14-day repeated oral administration of B-ZnO, N-ZnO or Zn ions. (**A**) The heat map, (**B**) dendrogram of hierarchical clustering, and (**C**) Venn diagram of differentially expressed genes (DEGs) satisfying with two-fold changes (>2 or <−2).

The gene expression profiles in the rat livers after repeated oral administration of ZnO particles (100 mg/kg) or an equivalent amount of Zn ions for 14 days were analyzed by next-generation sequencing. The heatmap of differentially expressed genes (DEGs) satisfying with two-fold changes (> 2 or < −2) was generated to visualize transcriptomic differences among B-ZnO, N-ZnO, Zn ions, and non-treated control. Up- and down-regulations were indicated as yellow and blue colors, respectively. Figure 5A clearly shows that the gene expression patterns of B-ZnO, N-ZnO or Zn ions-treated livers were remarkably different compared with that of non-treated control. Dendrogram of hierarchical clustering reveals a distinct difference in the expression patterns between ZnO particles and Zn ions (Figure 5B). Extremely low similarity of the gene expression pattern of Zn ions-treated liver compared with that of non-treated control was found. The total numbers of up- and down-regulated DEG satisfying with two-fold changes compared with non-treated control were 28, 34, and 60 for B-ZnO, N-ZnO, and Zn ions, respectively (Figure 5C). In particular, 14, 19, and 43 genes were differently expressed by only B-ZnO, N-ZnO or Zn ions, respectively, indicating that each administered material affects differently the gene expression profiles.

### 2.6. Identification of DEGs and Their Effect on Biological Functions

**Figure 6:**
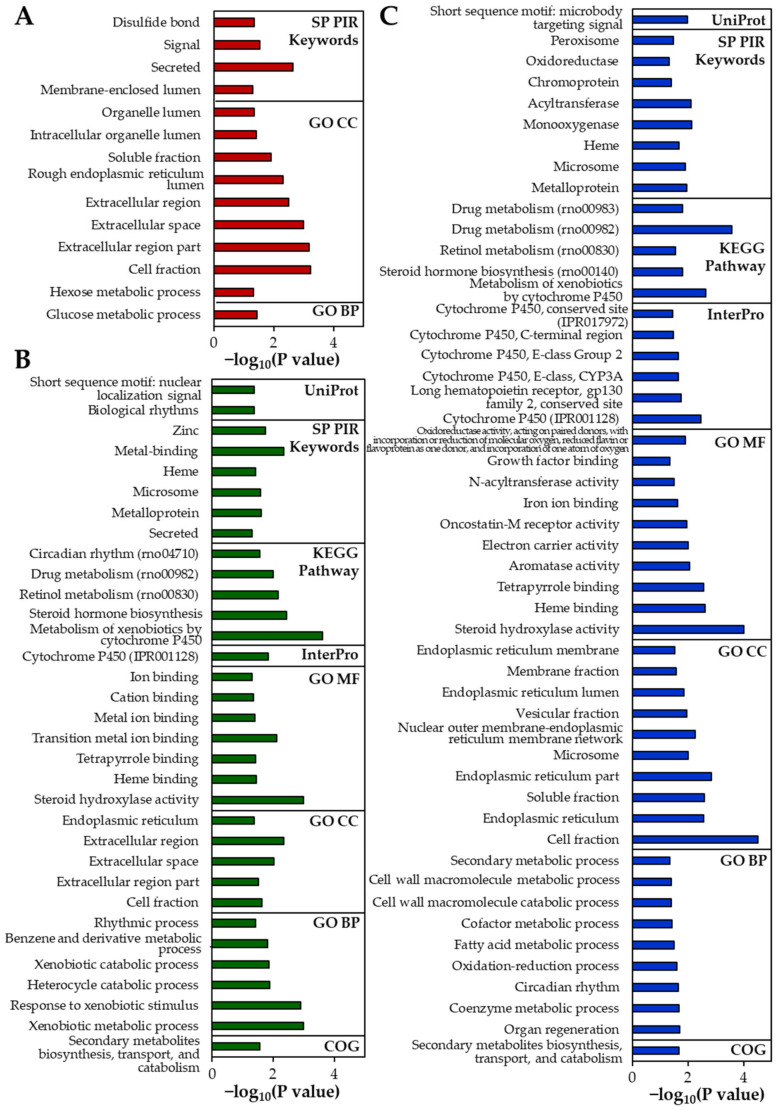
Gene enrichment analysis of the differentially expressed genes (DEGs) in the rat livers using Database for Annotation, Visualization, and Integrated Discovery (DAVID) tools after 14-day repeated oral administration of (**A**) B-ZnO, (**B**) N-ZnO or (**C**) Zn ions in rats. The significantly enriched terms are presented (modified Fisher Exact *p* < 0.05). SP PIR, Swiss-Prot and Protein Information Resource; KEGG, Kyoto encyclopedia of genes and genomes; GO, Gene Ontology; BP, Biological Process; CC, Cellular Component; MF, Molecular Function; COG, Cluster of Orthologous Groups.

The up-regulated DEGs identified in the rat livers are listed in Table 4. The numbers of up-regulated DEGs were determined to be 9, 16, and 34 for B-ZnO, N-ZnO, and Zn ions, respectively. The results of gene enrichment analysis also reveal a greater number of functional categories for Zn ions than for ZnO particles (Figure 6). In the Zn ions-treated liver, genes encoding cytochrome P450 (CYP450; *Cyp2b2*, *Cyp2c11*, *Cyp2c24*, and *Cyp3a18*) and metabolic process (*Csad* and *Fmo1*) [26,27] were markedly up-regulated compared with ZnO particles (Table 4). Not surprisingly, several functional categories involved in CYP450 and metabolism were significantly enriched in the rats administered Zn ions compared with ZnO particles (Figure 6). In addition, up-regulation of genes involved in tissue injury and hepatic fibrosis (*Adora1*) [28], caspase-dependent apoptosis (*Moap1*) [29], and oxidative stress and antioxidant defense (*Ephx2* and *Oxr1*) [30] was identified only in the liver treated with Zn ions (Table 4).

When particle sizes were compared, the up-regulation of CYP450-related genes (*Cyp1a1*, *Cyp3a2*, and *Cyp8b1*) and injury repair- or metabolic-related genes (*Akr1b8* and *Gsta3*) [26,31] was identified in the N-ZnO-treated liver, which was not observed in the B-ZnO-treated liver (Table 4). Accordingly, categories related to CYP450 or metabolism were clearly enriched in the rats administered N-ZnO compared with B-ZnO (Figure 6). The functional categories associated with metal ion binding were also remarkably identified in the liver treated with N-ZnO, which was not found in B-ZnO-treated liver (Figure 6). On the other hand, *Nr1d1*, a gene involved in the immune response [26], was upregulated only in the case of N-ZnO and Zn ions (Table 4).

Table 5 shows down-regulated DEGs in the ZnO particles- or Zn ions-treated rat livers compared with the non-treated control. Total down-regulated DEGs by B-ZnO, N-ZnO, and Zn ions, were 19, 18, and 26, respectively, indicating that Zn ions differently influenced on the gene expression compared with ZnO particles. Among them, 13, 13, and 19 of DEGs were down-regulated in the rat livers treated with only B-ZnO, N-ZnO, and Zn ions, respectively.

### 2.7. Protein-Protein Interaction Networks

**Figure 7:**
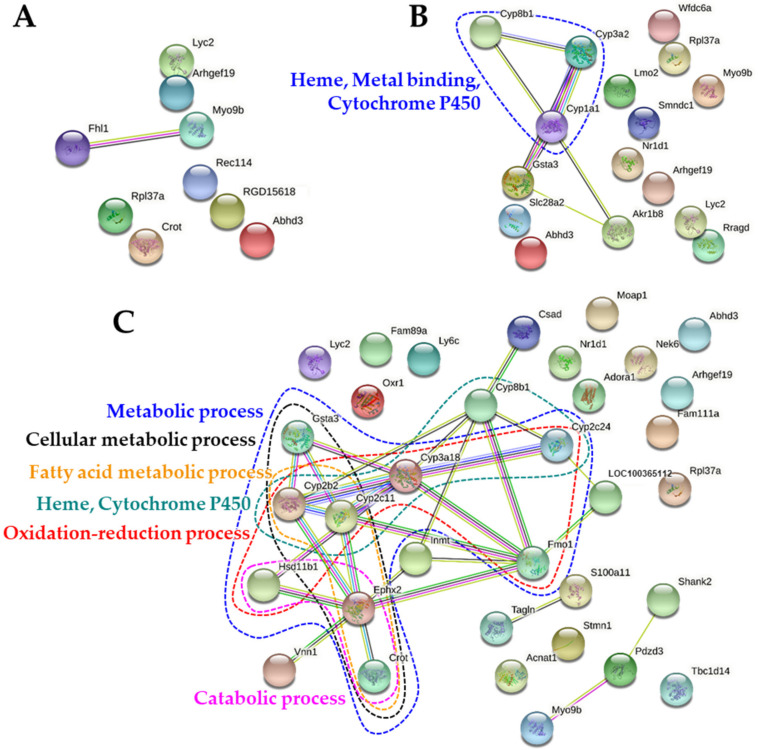
Protein-protein interaction (PPI) networks of up-regulated differentially expressed genes (DEGs) in the rat livers after 14-day repeated oral administration of (**A**) B-ZnO, (**B**) N-ZnO or (**C**) Zn ions.

**Figure 8:**
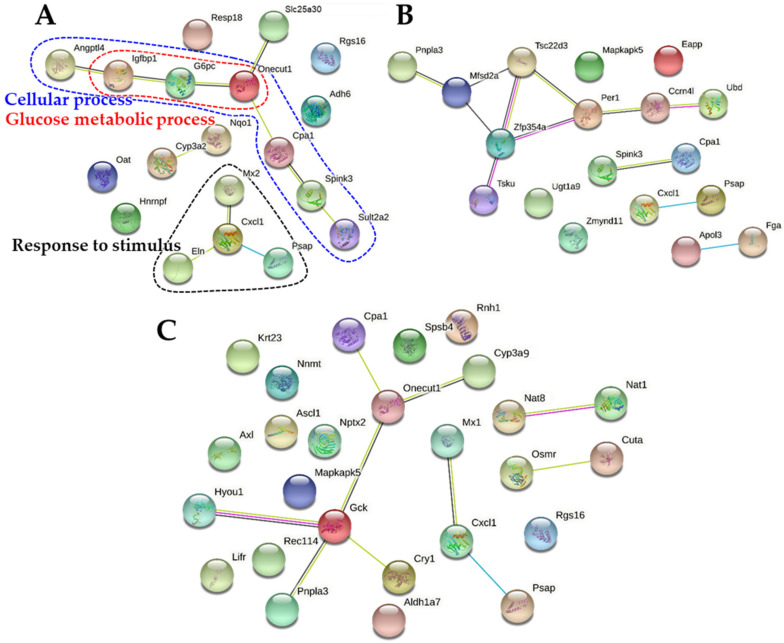
Protein-protein interaction (PPI) networks of down-regulated differentially expressed genes (DEGs) in the rat livers after 14-day repeated oral administration of (**A**) B-ZnO, (**B**) N-ZnO or (**C**) Zn ions.

The protein-protein interaction (PPI) networks of up-regulated and down-regulated DEGs were visualized using Search Tool for the Retrieval of Interacting Genes/Proteins (STRING) and are presented in Figure 7 and Figure 8, respectively. In the PPI networks of the up-regulated DEGs, no remarkable cluster was found by B-ZnO (Figure 7A), whereas the network associated with heme, metal binding, and CYP450 was observed by N-ZnO (Figure 7B), which is highly consistent with Table 4 and Figure 6. On the other hand, more PPI network clusters were found by Zn ions, mainly related to metabolic process including catabolic, oxidation-reduction, fatty acid metabolic, and cellular metabolic process (Figure 7C), which are also listed in Figure 6. In addition, the network related to heme and CYP450 was also found by Zn ions, as observed by N-ZnO (Figure 7C).

As shown in Figure 8A, the PPI networks related to cellular process and response to stimulus were down-regulated by B-ZnO, which were not observed in other treatments. No remarkably down-regulated PPI network correlated with down-regulated DEGs (Table 5) was clustered by N-ZnO and Zn ions (Figure 8B,C).

## 3. Discussion

The physicochemical properties of ZnO particles were investigated by SEM, DLS, and ELS. The SEM images and DLS data reveal that the constituent particle size and Z-average diameters of N-ZnO were quite different from those of B-ZnO (Supplementary Figure S1 and Table 1). Larger hydrodynamic diameters (Table 1) than constituent particle sizes (Supplementary Figure S1) also demonstrate that both ZnO particles formed aggregates under aqueous condition. It is worth noting that 5% glucose solution was used as a dispersant for all experiments, except zeta potential measurement, to enhance suspension stability of particles [32,33,34]. Indeed, ZnO easily tends to form agglomerates/aggregates and settles down in aqueous solutions [35]. On the other hand, as observed in the SEM image, N-ZnO can be classified as a nanosized material (<100 nm) because nanomaterials are defined as materials containing more than 50% of constituent particles of 1–100 nm in size [36]. These results suggest that N-ZnO is a nano-sized material, but present as an aggregate in aqueous solution. The zeta potential values of ZnO particles were found to be positive, regardless of particle size.

In vivo solubility in rat gastric fluid demonstrates that about 12% of ZnO particles could be dissolved after oral administration, regardless of particle size (Figure 1), suggesting that the dissolution property of ZnO particles was more influenced by biological environment than by particle size. This result is in good agreement with our previous ex vivo solubility (~11%) of ZnO particles in the gastric fluid [17,18]. On the other hand, in vitro dissolution properties of both B-ZnO and N-ZnO in simulated gastric fluid were reported to be ~25% [17,18]. This can be explained by the difference between in vitro and in vivo solubility test conditions; the rat gastric pH was reported to be ~3.2 [37], which is higher than that of the simulated gastric fluid (pH ~1.5). Hence, the higher pH of in vivo rat gastric fluid than that of in vitro simulated gastric fluid seems to contribute to lower in vivo solubility. It was also reported that the solubility is highly dependent on NP concentration [38,39]. Indeed, ZnO particles were added to in vitro simulated gastric fluids and ex vivo gastric extract at concentration of 5 and 4.5 mg/mL, respectively [17]. For in vivo solubility, the concentration of ZnO particles in the stomach can be calculated to be about 4 mg/mL, when considering the volume of gastric fluid for rats (3 mL) [37] after oral administration of 2 mL of ZnO particle suspension (10 mg/mL) in rats (based on a body weight of 200 g). Therefore, the concentrations among in vitro, ex vivo, and in vivo solubility experiments were similar and did not really induce different solubility.

Plasma concentration–time profiles suggest that Zn ions absorbed into the body faster than ZnO particles, and a large amount of Zn ions was absorbed compared with the particles (Figure 2). It should be noted that total Zn concentrations in Zn ions (zinc chloride), B-ZnO, and N-ZnO for oral administration were adjusted to the same. The toxicokinetic parameters also support these findings (Table 2). Indeed, T_max_ values of Zn ions (1.6 h) versus ZnO particles (~3.0 h) indicate more rapid absorption of Zn ions than particle forms. Remarkably higher C_max_, AUC, and absorption (%) values of Zn ions than those of ZnO particles imply high oral bioavailability of the former than the latters. Peak et al. reported that orally administered Zn ions were absorbed in a larger amount and faster than N-ZnO [40], which is in good agreement with our result. On the other hand, orally administered ZnO particles and Zn ions could be eliminated from the circulation system within 6 h after oral administration, based on low T_1/2_ and MRT values (Table 2), suggesting their low accumulation potential in the tissues. When we compare particle size, N-ZnO entered the bloodstream more massively than B-ZnO (Figure 2 and Table 2). It is worth noting that the major biological fate of ZnO particles is the particle form, as evidenced by in vivo solubility result (~12%) (Figure 1). Hence, the solubility of ZnO particles is not the only factor affecting their oral absorption. It is probable that ZnO particles can be also absorbed into the circulation system in particle forms. ZnO NPs can enter the bloodstream in a greater amount than micro-sized ZnO, probably related to its large specific surface area to volume ratio and high reactivity [9]. Lammel et al. reported that only NPs or their agglomerates of a specific size in the range of 30–100 nm were endocytosed, but larger agglomerates were excluded from the uptake [41]. It was also demonstrated that in vitro intestinal transport of polystyrene NPs using co-culture models was significantly higher than micro-sized particles (1 μm) [42]. Furthermore, several studies reported that NPs exhibited higher oral absorption than their bulk-sized counterparts [43,44,45]. These toxicokinetic results suggest that ZnO NPs may exhibit unpredictable biological responses after a prolonged exposure. These results also clearly indicate that toxicokinetic behaviors can be highly influenced by particle size and particle/ionic form of ZnO.

Repeated oral toxicity study for 14 days demonstrates no significant toxicity of ZnO particles or Zn ions in terms of changes in body weight, food intake, water consumption, and organo-somatic indices (Figure 3 and Table 3). Change in organo-somatic indices can be one of the important factors directly indicating the toxic effect of materials on target organs [46]. Moreover, no increased Zn levels in the plasma and rat organs treated with ZnO particles or Zn ions were found compared with non-treated control (Figure 4). This can be explained by low T_1/2_ and MRT values of ZnO particles or Zn ions (Table 2). Therefore, ZnO particles or Zn ions did neither cause acute oral toxicity nor accumulate in the target specific organs following 14-day repeated oral administration in rats.

However, the results of gene expressions in the rat livers following 14-day repeated oral administration clearly suggest that each administered material affects differently the gene expression profiles (Figure 5). In particular, the largest numbers of up- and down-regulated DEGs were found by Zn ions (Table 4 and Table 5). In the case of Zn ions, DEGs (Table 4), functional categories (Figure 6), and PPI networks (Figure 7) of up-regulated genes were primarily involved in CYP450 and metabolic process compared with ZnO particles. The CYP450 is a superfamily of hemeproteins that plays a role in the metabolism of drugs and other xenobiotics, facilitating the excretion and detoxification of substances through enzymatic conversion in the liver [47,48]. Hence, these results seem to be associated with the detoxification process of Zn ions massively absorbed (Figure 2 and Table 2). In addition, several up-regulation of genes related to tissue injury, hepatic fibrosis, and oxidative stress/antioxidant defense were also identified by Zn ions, suggesting their high toxicity potential and different toxicity mechanism compared with ZnO particles. 

In terms of particle size, small numbers of total up-and down-regulated genes were affected by B-ZnO compared with N-ZnO (Table 4 and Table 5). The enriched genes (Table 4), functional categories (Figure 6), and up-regulated PPI networks (Figure 7) associated with heme, metal binding, and CYP450 were clearly observed in the rat liver administered N-ZnO, which was not pronounced in the B-ZnO-treated liver. It is worth noting that these functions were attributed to *Cyp1a1, Cys3a2,* and *Cyp8b1* (Figure 7B), and among them, *Cyp1a1* and *Cys3a2* were up-regulated only in the N-ZnO-treated liver (Table 4). These results suggest that N-ZnO can cause more effect on transcriptomic expression than B-ZnO. Moreover, the potential toxicity and toxic mechanism of N-ZnO are probably related to the functions of heme, metal binding, and CYP450. In the N-ZnO-treated liver, up-regulation of *Nr1d1* was also observed, which was not found by B-ZnO. *Nr1d1* is known to be related to immune response [26]. In our previous proteomic results, complement C, which plays a crucial role in innate and adaptive immune response [49], was only identified in N-ZnO-plasma protein corona [17,18]. These findings suggest that repeated administration of N-ZnO is more likely to affect immune response than B-ZnO. It was reported that CYP1A1 was highly expressed in inflammatory macrophages and the mice skin, inducing release of pro-inflammatory mediators and immune cell activation [50,51]. Hence, signaling pathway involving inflammation response can be a target for further mechanistic toxicity study. On the other hand, the functional categories of metal ion binding and metalloprotein were more affected by N-ZnO compared with B-ZnO or Zn ions (Figure 6 and Figure 7). It is known that cysteine-rich metal-binding proteins like metallothionein are known to be involved in the uptake and distribution of Zn ions [52,53]. Our previous study showed that new Zn-S bonds, determined by X-ray absorption spectroscopy analysis, were found in the liver, kidney, and spleen after oral administration of ZnO NPs in rats. These findings suggest that ZnO NPs could be dissolved in the liver or during uptake, and form Zn-S bonds probably with metal-binding proteins [54]. Therefore, distribution and biological fate of N-ZnO in the organs seem to be mainly mediated by metal binding-proteins.

In terms of down-regulated DEGs, the PPI networks associated with cellular process and response to stimulus were found by B-ZnO, whereas other functional clusters were not remarkably observed by N-ZnO or Zn ions (Figure 8). Thus, the possibility that B-ZnO also causes the toxicity cannot be completely excluded, although its toxic mechanism seems to be different from those of N-ZnO or Zn ions. Meanwhile, all the results obtained by Zn ions were completely different from those by ZnO particles. The potential toxicity of Zn ions is primarily associated with up-regulation of metabolic process (Figure 6 and Figure 7C). Zn ions have the potential to induce more toxicity than B-ZnO and N-ZnO based on the total number of DEGs (Table 4 and Table 5), probably related to high oral absorption (Figure 2 and Table 2). Taken together, the transcriptomic results clearly show that particle size and particle/ionic forms of ZnO could influence differently the mechanistic biological functions. N-ZnO is expected to have lower toxicity than Zn ions, but it may cause unexpected toxicological effects compared with B-ZnO after a long-term exposure. A subchronic toxicity study followed by signaling pathway, transcriptomic, and proteomics analysis in tissues is required to assume the toxicity of ZnO NPs.

## 4. Materials and Methods

### 4.1. Materials and Chemicals

B-ZnO (<5 µm) and N-ZnO (<100 nm) were purchased from Sigma-Aldrich (St. Louis, MO, USA). ZnO particles were dispersed in 5% glucose (Sigma-Aldrich) for 30 min followed by sonication (Bransonic 5800, Branson Ultrasonics, Danbury, CT, USA) for 5 min, prior to experiments. An equivalent amount of Zn ion (Sigma-Aldrich) solution based on Zn content was prepared in the same manner and used for comparative study. Heparin was supplied by Sigma-Aldrich. Nitric acid (HNO_3_), and hydrogen peroxide (H_2_O_2_) were bought from Samchun Pure chemical Co., Ltd. (Pyeongtaek, Gyeonggi-do, Republic of Korea). RNAeasy mini kit was obtained from Qiagen (Hilden, Germany).

### 4.2. Characterization

The constituent particle size and shape of each ZnO particle were observed by SEM (JSM-7100F, JEOL, Tokyo, Japan). The average particle sizes and size distributions were measured by randomly selecting 200 particles from the SEM images. Zeta potentials and hydrodynamic diameters of ZnO particles were determined by Zetasizer Nano System (Malvern Instruments, Worcestershire, UK).

### 4.3. Animals

Six-week-old female Sprague Dawley (SD, 170–180 g) rats were obtained from Koatech. Co. (Pyeongtaek, Gyeonggi-do, Republic of Korea). Rats were housed in plastic laboratory animal cages on a ventilated rack, maintained at 20 ± 2 °C and 60 ± 10% relative humidity under a 12 h light/dark cycle. Commercial laboratory complete food and water were given to the animals ad libitum. Rats were acclimated to the experimental environment for 7 days before administration. All animal experiments were performed in compliance with the guideline issued by the Animal and Ethics Review Committee of Seoul Women’s University (IACUC-2014A-2), Republic of Korea.

### 4.4. In Vivo Dissolution Properties of ZnO Particles

Rats were orally administered a single-dose of 100 mg/kg ZnO particles dispersed in 5% glucose. After 15 min, the gastric fluids in the stomach were collected and centrifuged at 16,000× *g* for 15 min at 4 °C to obtain the supernatants. The dissolved Zn contents from ZnO particles in the supernatants were analyzed using ICP-AES (JY2000 Ultrace, HORIBA Jobin Yvon, Longjumeau, France) after pre-digestion with HNO_3_ and H_2_O_2_ as described in “ICP-AES analysis”.

### 4.5. Toxicokinetic Study

Rats were orally administered a single-dose of 100 mg/kg ZnO particles dispersed in 5% glucose. An equivalent amount of Zn ion solution based on Zn content was also administered in rats for comparative study. Blood samples were collected from tail veins at 0, 0.25, 0.5, 1, 2, 4, 6, 10, and 24 h after administration, and centrifuged at 3000× *g* for 15 min at 4 °C to obtain plasma. The Zn concentrations were determined by ICP-AES following pre-digestion with HNO_3_ and H_2_O_2_ as described in “ICP-AES analysis”. Toxicokinetic parameters including maximum concentration (C_max_), time to maximum concentration (T_max_), area under the plasma concentration–time curve (AUC), half-life (T_1/2_), and mean residence time (MRT) were estimated using Kinetica software (version 4.4, Thermo Fisher Scientific, Waltham, MA, USA).

### 4.6. 14-Day Repeated Oral Toxicity and Tissue Accumulation

Rats were orally administered 100 mg/kg ZnO particles dispersed in 5% glucose or an equivalent amount of Zn ions solution based on Zn content for 14 consecutive days. An equal volume of 5% glucose was administered to the non-treated control in the same manner. Changes in body weights, behaviors, specific symptoms, and food or water consumptions were daily examined and recorded after administration. At the end of experiment, animals were sacrificed by carbon dioxide euthanasia and organs (kidney, liver, lung, and spleen) were collected. Organo-somatic index was calculated by the following formula: weight of the organ (g)/total body weight (g) × 100. Zn contents in the tissues were determined as described “ICP-AES analysis”.

### 4.7. ICP-AES Analysis

The samples were digested with 10 mL of HNO_3_ and 1 mL of H_2_O_2_ at 200 °C, and this procedure was repeated until the solutions were colorless and completely evaporated. Total Zn concentrations were analyzed by ICP-AES after dilution to 10 mL with deionized and distilled water.

### 4.8. RNA Extraction for Sequencing

The liver samples from 3 replicates were pooled in an equal quantity after daily oral administration of ZnO particles (100 mg/kg) or an equivalent amount of Zn ions for 14 days in rats. Total RNA was isolated from the pooled livers using the RNAeasy mini kit according to the manufacturer’s protocol. Total RNA concentrations were calculated by Quant-IT RiboGreen (Invitrogen, Carlsbad, CA, USA). Only high-quality RNA with RNA integrity number greater than 7.0, obtained by the TapeStation RNA ScreenTape (Agilent Technologies, Santa Clara, CA, USA), was used for library construction.

### 4.9. RNA Sequencing and Data Analysis

A cDNA library was constructed with 1 μg of total RNA for each sample by Illumina TruSeq RNA Library Preparation Kit (Illumina, Inc., San Diego, CA, USA) and sequenced in a paired-end mode using the HiSeq 2500 platform in an 101 bp read length by the Macrogen Inc. (Seoul, Republic of Korea). Raw reads were trimmed using Trimmomatic (version 0.32) (http://www.usadellab.org/cms/?page=trimmomatic), and mapped against the genome of UCSC rn4 genome browser using TopHat (version 2.0.13) (http://ccb.jhu.edu/software/tophat/index.shtml). The mapped reads were then assembled with Cufflinks (version 2.2.1) (http://cole-trapnell-lab.github.io/cufflinks/) and calculated into fragments per kilobase of transcript, per million mapped reads (FPKM). For analysis of DEGs, 12,296 transcripts were used from a total of 17,915 transcripts because transcripts with FPKM values of zero in at least one sample were excluded. At least two-fold changes were used as the criteria for differential expression. Positively and negatively changed values indicate up-regulation and down-regulation, respectively. Hierarchical clustering analysis was performed using Euclidean distance to identify similarity of DEG patterns among non-treated control and sample-treated groups.

### 4.10. Gene-Set Enrichment Analysis and PPI Networks RNA Sequencing and Data Analysis

Gene enrichment analysis based on functional annotation database was performed using the DAVID tool to investigate whether the differences in DEGs affect biological functions [55]. The gene term annotation referenced in the DAVID tool is based on different annotation categories (Gene Ontology (GO), Biological Process (BP), GO Cellular Component (CC), GO Molecular Function (MF), Kyoto encyclopedia of genes and genomes (KEGG) Pathways, InterPro, Cluster of Orthologous Groups (COG), Swiss-Prot and Protein Information Resource (SP PIR) Keywords, and UniProt). The significant enriched categories were determined by Expression Analysis Systematic Explorer score (modified Fisher Exact *p* < 0.05) associated with the enriched annotation terms that belong to the gene group. The PPI networks were investigated with STRING (version 11.0) (http://string-db.org/).

### 4.11. Statistical Analysis

All results were expressed as means ± standard deviations. Statistical analysis was performed using one-way analysis of variance (ANOVA) with Tukey’s test in SAS version 9.4 (SAS Institute Inc., Cary, NC, USA) to determine the significances of intergroup differences. Statistical significance was accepted for *p* values of less than 0.05.

## 5. Conclusions

In this study, the effects of particle size and biological fate of ZnO on acute toxicity, toxicokinetics, and gene expression profiles in the livers were evaluated following oral administration of B-ZnO, N-ZnO or Zn ions in rats. The in vivo solubility of ZnO particles was found to be 12%, regardless of the particle size, suggesting that the major biological fate of ZnO particles was the particle form. After a single-dose oral administration, high oral absorption of Zn ions, followed by N-ZnO and B-ZnO were found, indicating different toxicokinetic behaviors depending on particle/ionic forms and particle size. Transcriptomic expressions in the livers were differently affected after repeated oral administration in rats for 14 days, showing up-regulation of metabolic process and metal binding by Zn ions and N-ZnO, respectively, and down-regulation of cellular process by B-ZnO, despite no significant acute toxicity found in all cases. It can be, therefore, concluded that unexpected toxicological responses could be induced depending on particle size and fate of ZnO after a long-term exposure and more caution should be taken for food additive ZnO NPs.

## Figures and Tables

**Table 1:** Particle fractions, hydrodynamic diameters, and surface charges of ZnO particles.

ZnO Particle	Fraction (Number %)			Fraction (Mass %)			Z-Average Diameter (nm)	Zeta Potential (mV)
	<100 nm	100–200 nm	>200 nm	<100 nm	100–200 nm	>200 nm		
B-ZnO	ND ^1^	ND ^1^	100.0 ± 0.0	ND ^1^	ND ^1^	100.0 ± 0.0	603.6 ± 1.9 ^a^	9.5 ± 0.4 ^a^
N-ZnO	ND ^1^	9.3 ± 5.1	90.7 ± 5.1	ND ^1^	1.4 ± 0.6	98.6 ± 0.6	401.1 ± 4.2 ^b^	12.8 ± 0.2 ^b^

^a^ and ^b^ indicate significant differences between B-ZnO and N-ZnO (*p* < 0.05). ^1^ Not detectable.

**Table 2:** Toxicokinetic parameters and oral absorption after a single-dose oral administration of B-ZnO, N-ZnO or Zn ions in rats.

Toxicokinetic Parameter ^1^	B-ZnO	N-ZnO	Zn Ions
C_max_ (µg/mL)	16.6 ± 2.8 ^a^	44.8 ± 8.5 ^b^	132.0 ± 37.6 ^c^
T_max_ (h)	2.8 ± 1.1 ^a^	3.0 ± 1.2 ^a^	1.6 ± 0.7 ^b^
AUC (h × µg/mL)	70.8 ± 2.7 ^a^	212.4 ± 24.0 ^b^	450.6 ± 77.7 ^c^
T_1/2_ (h)	3.2 ± 1.1 ^a^	3.3 ± 1.4 ^a^	3.3 ± 0.5 ^a^
MRT (h)	4.1 ± 0.7 ^a^	4.6 ± 1.3 ^a^	5.8 ± 0.9 ^a^
Absorption (%)	5.0 ± 0.1 ^a^	14.5 ± 1.6 ^b^	31.4 ± 5.4 ^c^

^a^, ^b^, and ^c^ indicate significant differences among B-ZnO, N-ZnO, and Zn ions (*p* < 0.05). ^1^ C_max_, maximum concentration; T_max_, time to maximum concentration; AUC, area under the plasma concentration-time curve; T_1/2_, half-life; MRT, mean residence time.

**Table 3:** Organo-somatic indices of rats after 14-day repeated oral administration of B-ZnO, N-ZnO or Zn ions ^1^.

Organ	Control	B-ZnO	N-ZnO	Zn Ions
Kidney	1.0 ± 0.1	1.1 ± 0.0	1.0 ± 0.1	1.1 ± 0.0
Liver	3.8 ± 0.5	4.3 ± 0.3	4.2 ± 0.5	4.1 ± 0.2
Lung	0.6 ± 0.1	0.6 ± 0.0	0.6 ± 0.0	0.6 ± 0.1
Spleen	0.2 ± 0.0	0.3 ± 0.0	0.3 ± 0.0	0.3 ± 0.0

^1^ No significant differences among non-treated control and treated groups (B-ZnO, N-ZnO, and Zn ions) were found (*p* > 0.05).

**Table 4:** Two-fold up-regulated differentially expressed genes (DEGs) compared with non-treated control in the livers after 14-day repeated oral administration of B-ZnO, N-ZnO or Zn ions in rats.

Gene	Gene Description	Fold Change		
		B-ZnO	N-ZnO	Zn Ions
*Abhd3*	Abhydrolase domain containing 3	2.13	2.23	2.42
*Acnat2*	Acyl-coenzyme A amino acid N-acyltransferase 2			2.33
*Adora1*	Adenosine A1 receptor			2.18
*Akr1b8*	Aldo-keto reductase family 1, member B8		2.09	
*Arhgef19*	Rho guanine nucleotide exchange factor (GEF) 19	2.52	2.90	2.96
*Crot*	Carnitine O-octanoyltransferase	3.32		2.06
*Csad*	Cysteine sulfinic acid decarboxylase			2.25
*Cyp1a1*	Cytochrome P450, family 1, subfamily a, polypeptide 1		3.02	
*Cyp2b2*	Cytochrome P450, family 2, subfamily b, polypeptide 2			2.22
*Cyp2c11*	Cytochrome P450, subfamily 2, polypeptide 11			2.40
*Cyp2c24*	Cytochrome P450, family 2, subfamily c, polypeptide 24			2.60
*Cyp3a2*	Cytochrome P450, family 3, subfamily a, polypeptide 2		2.62	
*Cyp3a18*	Cytochrome P450, family 3, subfamily a, polypeptide 18			2.02
*Cyp8b1*	Cytochrome P450, family 8, subfamily b, polypeptide 1		2.55	2.18
*Ephx2*	Epoxide hydrolase 2, cytoplasmic			2.03
*Fam111a*	Family with sequence similarity 111, member A			2.13
*Fam89a*	Family with sequence similarity 89, member A			2.36
*Fhl1*	Four and a half LIM domains 1	2.12		
*Fmo1*	Flavin containing monooxygenase 1			2.34
*Gsta3*	Glutathione S-transferase alpha 3		2.86	2.06
*Hsd11b1*	Hydroxysteroid 11-beta dehydrogenase 1			2.00
*Inmt*	Indolethylamine N-methyltransferase			2.55
*Lmo2*	LIM domain only 2		2.39	
*LOC100365112*	Carboxylesterase-like precursor			2.47
*Ly6c*	Ly6-C antigen			2.15
*Lyc2*	Lysozyme C type 2	2.69	2.66	2.15
*Moap1*	Modulator of apoptosis 1			3.10
*Myo9b*	Myosin IXb	3.74	2.10	2.23
*Nek6*	NIMA-related kinase 6			3.53
*Nr1d1*	Nuclear receptor subfamily 1, group D, member 1		2.24	2.15
*Oxr1*	Oxidation resistance 1			2.10
*Pdzd3*	PDZ domain containing 3			2.21
*Rec114*	REC114 meiotic recombination protein	2.37		
*RGD15618*	Similar to RIKEN cDNA 3110035E14	2.06		
*Rpl37a*	Ribosomal protein L37a	5.95	4.01	11.54
*Rragd*	Ras-related GTP binding D		2.07	
*S100a11*	S100 calcium binding protein A11			2.04
*Shank2*	SH3 and multiple ankyrin repeat domains 2			2.03
*Slc28a2*	Solute carrier family 28 (sodium-coupled nucleoside transporter), member 2		2.42	
*Smndc1*	Survival motor neuron domain containing 1		2.29	
*Stmn1*	Stathmin 1			2.20
*Tagln*	Transgelin			2.01
*Tbc1d14*	TBC1 domain family, member 14			2.13
*Vnn1*	Vanin 1			2.72
*Wfdc6a*	WAP four-disulfide core domain 6A		2.10	

**Table 5:** Two-fold down-regulated differentially expressed genes (DEGs) compared with non-treated control in the livers after 14-day repeated oral administration of B-ZnO, N-ZnO or Zn ions in rats.

Gene	Gene Description	Fold Change		
		B-ZnO	N-ZnO	Zn Ions
*Adh6*	Alcohol dehydrogenase 6 (class V)	−4.20		
*Aldh1a7*	Aldehyde dehydrogenase, cytosolic 1			−2.46
*Angptl4*	Angiopoietin-like 4	−2.59		
*Apol3*	Apolipoprotein L3		−2.09	
*Ascl1*	Achaete-scute family bHLH transcription factor 1			−2.24
*Axl*	Axl receptor tyrosine kinase			−2.26
*Ccrn4l*	CCR4 carbon catabolite repression 4-like (S. cerevisiae)		−2.35	
*Cpa1*	Carboxypeptidase A1 (pancreatic)	−2.57	−3.03	−2.86
*Cry1*	Cryptochrome circadian clock 1			−2.08
*Cuta*	CutA divalent cation tolerance homolog (E. coli)			−2.49
*Cxcl1*	Chemokine (C-X-C motif) ligand 1	−2.90	−3.03	−2.77
*Cyp3a2*	Cytochrome P450, family 3, subfamily a, polypeptide 2	−2.18		
*Cyp3a9*	Cytochrome P450, family 3, subfamily a, polypeptide 9			−2.38
*Eapp*	E2F-associated phosphoprotein		−2.07	
*Eln*	Elastin	−2.17		
*Fga*	Fibrinogen alpha chain		−2.16	
*G6pc*	Glucose-6-phosphatase, catalytic subunit	−4.34		
*Gck*	Glucokinase			−2.08
*Hnrnpf*	Heterogeneous nuclear ribonucleoprotein F	−2.13		
*Hyou1*	Hypoxia up-regulated 1			−2.42
*Igfbp1*	Insulin-like growth factor binding protein 1	−2.17		
*Krt23*	Keratin 23 (histone deacetylase inducible)			−2.82
*Lifr*	Leukemia inhibitory factor receptor alpha			−3.52
*Mapkapk5*	Mitogen-activated protein kinase-activated protein kinase 5		−3.10	−2.19
*Mfsd2a*	Major facilitator superfamily domain containing 2A		−2.55	
*Mx1*	Myxovirus (influenza virus) resistance 1			−2.00
*Mx2*	Myxovirus (influenza virus) resistance 2	−2.19		
*Nat1*	N-acetyltransferase 1 (arylamine N-acetyltransferase)			−2.13
*Nat8*	N-acetyltransferase 8			−2.07
*Nnmt*	Nicotinamide N-methyltransferase			−2.16
*Nptx2*	Neuronal pentraxin Ⅱ			−2.40
*Nqo1*	NAD(P)H dehydrogenase, quinone 1	−2.14		
*Oat*	Ornithine aminotransferase	−2.10		
*Onecut1*	One cut homeobox 1	−2.06		−2.28
*Osmr*	Oncostatin M receptor			−2.48
*Per1*	Period circadian clock 1		−2.38	
*Pnpla3*	Patatin-like phospholipase domain containing 3		−2.03	−2.29
*Psap*	Prosaposin	−2.04	−2.04	−2.04
*Rec114*	REC114 meiotic recombination protein			−2.15
*Resp18*	Regulated endocrine-specific protein 18	−2.56		
*Rgs16*	Regulator of G-protein signaling 16	−5.87		−2.67
*Rnh1*	Ribonuclease/angiogenin inhibitor 1			−2.02
*Slc25a30*	Solute carrier family 25, member 30	−2.15		
*Spink3*	Serine peptidase inhibitor, Kazal type 3	−2.08	−2.28	
*Spsb4*	SplA/ryanodine receptor domain and SOCS box containing 4			−2.06
*Sult2a2*	Sulfotransferase family 2A, dehydroepiandrosterone (DHEA)-preferring, member 2	−2.11		
*Tsc22d3*	TSC22 domain family, member 3		−2.02	
*Tsku*	Tsukushi, small leucine rich proteoglycan		−2.03	
*Ubd*	Ubiquitin D		−2.04	
*Ugt1a9*	UDP glucuronosyltransferase 1 family, polypeptide A9		−2.35	
*Zfp354a*	Zinc finger protein 354A		−3.23	
*Zmynd11*	Zinc finger, MYND-type containing 11		−3.12	

## Data Availability

The data presented in this study are available in the article and Supplementary Information.

## Supplementary Materials

The following are available online at https://www.mdpi.com/1422-0067/22/4/1698/s1, Figure S1: scanning electron microscopy (SEM) images and size distributions of (A) B-ZnO and (B) N-ZnO. Particle size distributions were determined by randomly selecting 200 particles from the SEM images.

## Abbreviations

ANOVA	analysis of variance
AUC	area under the plasma concentration-time curve
BP	Biological Process
B-ZnO	bulk-sized zinc oxide
CC	Cellular Component
C_max_	maximum concentration
COG	Cluster of Orthologous Groups
CYP450	cytochrome P450
DAVID	Database for Annotation, Visualization, and Integrated Discovery
DEG	differentially expressed gene
DLS	dynamic light scattering
ELS	electrophoretic light scattering
FPKM	fragments per kilobase of transcript, per million mapped reads
GI	gastrointestinal
GO	Gene Ontology
H_2_O_2_	hydrogen peroxide
HNO_3_	nitric acid
ICP-AES	inductively coupled plasma–atomic emission spectroscopy
KEGG	Kyoto encyclopedia of genes and genomes
LOAEL	lowest-observed-adverse-effect level
MF	Molecular Function
MRT	mean residence time
NP	nanoparticle
N-ZnO	zinc oxide nanoparticle
PPI	protein-protein interaction
SD	Sprague Dawley
SEM	scanning electron microscopy
SP PIR	Swiss-Prot and Protein Information Resource
STRING	Search Tool for the Retrieval of Interacting Genes/Proteins
T_1/2_	half-life
T_max_	time to maximum concentration
Zn	zinc
ZnO	zinc oxide
