# Supplementary material for: Particle Size and Biological Fate of ZnO Do Not Cause Acute Toxicity, but Affect Toxicokinetics and Gene Expression Profiles in the Rat Livers after Oral Administration
Source: Int J Mol Sci. 2021 Feb 8;22(4):1698. doi: 10.3390/ijms22041698 (PMC7915389; doi:10.3390/ijms22041698)
Supplement: Supplementary file 1 [file ijms-22-01698-s001.pdf]

Particle Size and Biological Fate of ZnO Do Not Cause Acute Toxicity, but Affect Toxicokinetics and Gene Expression Profiles in the Rat Liver after Oral Administration

Jin Yu and Soo-Jin Choi *

Division of Applied Food System, Major of Food Science & Technology, Seoul Women's University, Seoul 01797, Korea; ky5031@swu.ac.kr (J.Y.)

* Correspondence: sjchoi@swu.ac.kr; Tel.: +82-2-970-5634; Fax: +82-2-970-5977

Received: 6 January 2021; Accepted: 5 February 2021; Published: 8 February 2021

Supplementary Materials

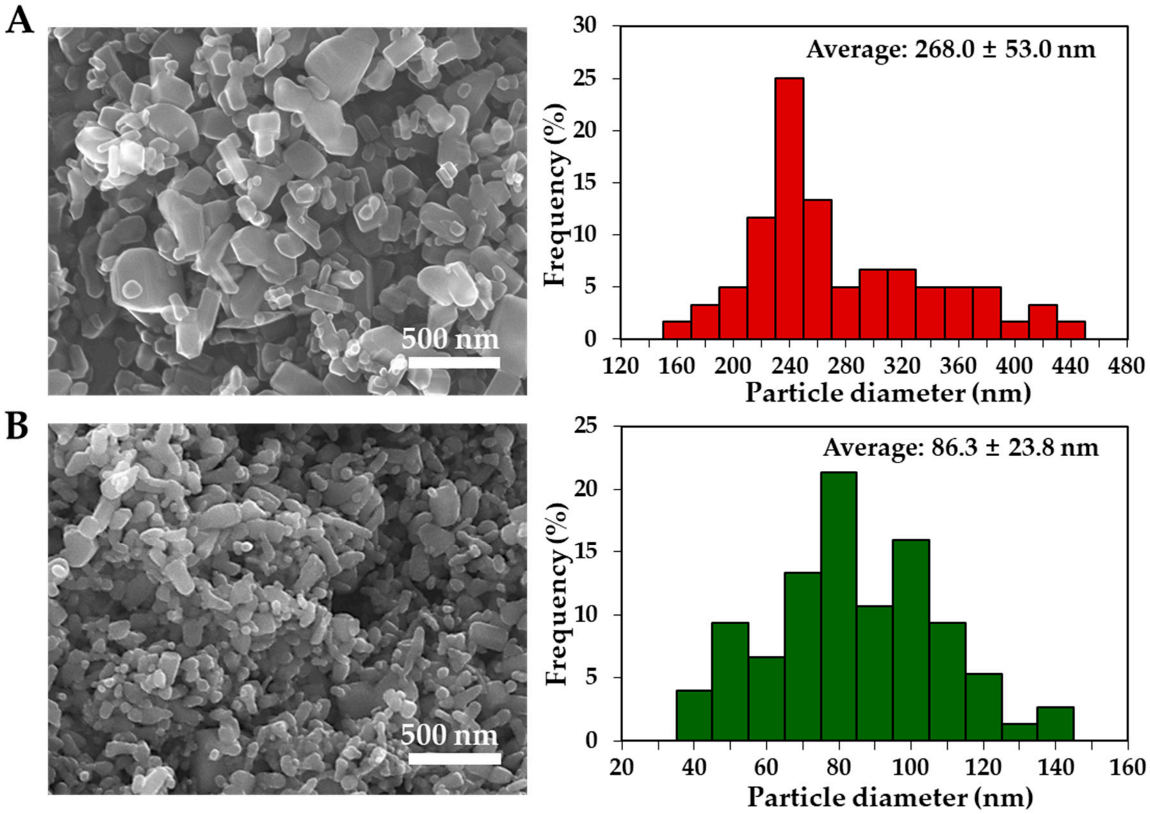

Figure 1. Scanning electron microscopy (SEM) images and size distributions of (**A**) B-ZnO and (**B**) N-ZnO. Particle size distributions were determined by randomly selecting 200 particles from the SEM images.
